# Initial Treatment for Unruptured Intracranial Aneurysm and Its Follow-up: A Cost Analysis of Pipeline Flow Diverters versus Coiling

**DOI:** 10.7759/cureus.5692

**Published:** 2019-09-18

**Authors:** Spencer Twitchell, Herschel W Wilde, Philipp Taussky, Michael Karsy, Ramesh Grandhi

**Affiliations:** 1 Neurosurgery, University of Utah School of Medicine, Salt Lake City, USA

**Keywords:** aneurysms, value-driven outcomes, cost analysis, follow-up, coiling, pipeline, pipeline embolization device

## Abstract

Purpose

Intracranial aneurysms are relatively common epidemiological problems for which the surveillance, treatment, and follow-up are costly. Although multiple studies have evaluated the treatment cost of aneurysms, the follow-up costs are often not examined. In our study, we analyzed how follow-up costs after treatment affected the overall cost of different endovascular techniques for treating aneurysms.

Materials and methods

An institutional database was used to evaluate the upfront and follow-up costs incurred by patients who underwent elective coiling or placement of a pipeline embolization device (PED) for the treatment of unruptured intracranial aneurysms from July 2011 to December 2017.

Results

A total of 114 patients (coiling, n = 37; PED, n = 77 ) were included in the study. There was no significant difference among patients in mean age [61.3 (±12.8 years) vs. 57.0 (±14.5 years); probability value (p) = 0.2], sex (male: 32.4% vs. 22.1%; p = 0.2), American Society of Anesthesiologists (ASA) grade (p = 0.5), discharge disposition (p = 0.1), mean length of stay [3.1 days (±5.5) vs. 2.4 days (±2.6); p = 0.2) or follow-up period [22.7 months (±18.5) vs. 18.6 months (±14.9); p = 0.2). There were no differences in costs during admission (p = 0.5) or in follow-up (p = 0.3) between coiling and PED treatments. Initial costs were predominantly related to supplies/implants (56.1% vs. 63.7%) for both treatments. Follow-up costs mostly comprised facility costs (68.2% vs. 67.5%), and there were no differences in costs of subgroups such as supplies/implants (10.5% vs. 9.4%), imaging (17.0% vs. 17.8%), or facilties between coiling and PED.

Conclusion

These results suggest that the upfront and follow-up costs are mostly similar for the treatment of intracranial aneurysms irrespective of whether the providers used coiling or PED endovascular techniques. Hence, we conclude that follow-up costs should not be a deciding factor when considering these treatments.

## Introduction

Intracranial aneurysms are a common intracranial disease, with a prevalence of 1-8% in the US and an approximate annual rupture risk of 1% [1]. Intracranial aneurysms at a higher risk of ruptures are traditionally treated with craniotomy and clipping. But the development of catheter-based endovascular techniques has significantly altered the treatment of intracranial aneurysms in modern healthcare. The outcomes of numerous studies, including the Barrow Ruptured Aneurysm Trial (BRAT) [2], Analysis of Treatment by Endovascular Approach of Non-ruptured Aneurysms (ATENA) study [3], Cerebral Aneurysm Rerupture After Treatment (CARAT) study [4], Clinical and Anatomical Results in the Treatment of Ruptured Intracranial Aneurysms (CLARITY) study [5], and international subarachnoid aneurysm trial (ISAT) [6] have transformed the approach towards managing patients with aneurysms. Additional advancements in neurointerventional technologies include the advent of the pipeline embolization device (PED) [7-10], development of stents and balloons as adjunctive devices for coil embolization of aneurysms, and availability of other wide-neck occlusion devices [11]. These studies have improved the variety of tools used to treat patients with aneurysms regardless of whether the providers use open or endovascular approaches. Despite these developments, intracranial aneurysms remain difficult to treat and manage. The common challenges are the significant intraprocedural complication risk as well as the potential for perioperative and delayed postoperative complications. Patients also require extensive follow-up via serial imaging to monitor aneurysm growth, residual, and other complications [10,12,13].

Various endovascular treatment modalities for aneurysms have been comparatively analyzed for efficacy, complication rates, and patient costs [7,8,14-17]. Although previous studies have sought to evaluate the total direct patient cost of the initial treatment, significant limitations exist in understanding the follow-up costs and their impact on total lifetime-treatment cost [16,18]. We aimed to compare both the direct treatment and the follow-up costs incurred by patients after they underwent coiling or flow diversion with PED by using the Values Driven Outcomes (VDO) database, a proprietary cost database (University of Utah Health, Salt Lake City, UT). We hypothesized that despite the greater upfront cost of treatment with PEDs, a lower follow-up cost would be seen in comparison to coiling.

## Materials and methods

Patient population

Before data collection, the Institutional Review Board (IRB) granted approval with a waiver of consent. Patients initially treated from July 2011 to January 2017 were identified from our institutional database. The inclusion criteria were as follows: elective cases without aneurysm rupture, treatment with coiling or PED, availability of clinical variables, and at least 1 follow-up after the treatment. Patients were identified in the database by Common Procedural Terminology (CPT) codes or International Classification of Disease, Tenth Edition (ICD10) codes, primarily the CPT code 61624 (endovascular therapy procedures on skull, meninges, and brain). Patients were also cross-referenced to a prospectively maintained database of endovascularly treated aneurysms. Coiling treatments considered included any embolization performed with stent- or balloon-assisted techniques as well as those performed with primary coiling alone. Follow-up imaging and laboratory studies (e.g., P2Y12) were done not based on any standardized protocol but left to the discretion of the treating physician. To verify that patients had unruptured, endovascularly treated aneurysms with adequate recorded data, a retrospective chart review of the electronic medical record was performed. Age, sex, aneurysm location, American Society of Anesthesiologists (ASA) physical status classification system, discharge disposition, length of stay, follow-up length, and costs were collected. The follow-up-length metric was further subdivided into two sections: the date of the last clinical encounter and the date of the last imaging evaluation.

Analysis

An institutional database was used to extract direct initial treatment costs as well as follow-up costs of the aneurysm treatment. This database is a tool designed to assess the direct cost of treatment per patient based on hospital costs [19]. Total costs and subcategory (pharmacy, supplies and implants, laboratory, imaging, and facility) costs were obtained for the initial treatment. All endovascular-related follow-up costs were summed up to generate a follow-up cost subtotal. As per our agreement with the institution, actual dollar amounts were not reported. A mean percentage of the total cost was generated as an alternative to presenting the actual cost, factoring each patient’s contribution to the total cost of the cohort. Individual costs were totaled for the cohort of patients and the contribution of each patient to the total cost was calculated, allowing for standard deviations, patient totals, subgroup costs, and means to be compared. Subcategory costs were reported as a percentage of the total cost and summed up to 100%.

The standard deviations of the mean (±) or absolute numbers with the percentage of the total were reported as appropriate. A probability value (p) of <0.05 was considered statistically significant. All statistical analysis was done using IBM SPSS Statistics (V20.0, IBM, Armonk, New York).

## Results

Patient characteristics

A total of 176 patients with elective aneurysms treated by coiling or PEDs were identified. Of these, 114 (coiling, n = 37; PED, n = 77) patients were included in the final analysis that included adequate follow-up information and cost data (Table 1). The patients in the PED group had a total of 79 aneurysms treated. The data relating to the mean age [61.3 (±12.8 years) vs. 57.0 (±14.5 years); p = 0.2] and sex (males: 32.4% vs. 22.1%; p = 0.2) were not significantly different between the coiling and PED groups. There were significant differences in the aneurysm location (p = 0.0001). Coiling was most commonly used when the locations of aneurysms were anterior communicating artery (n = 11) and internal carotid artery (n = 7), whereas PED was most common for aneurysms in the internal carotid artery (n = 51) and vertebral/posterior circulation (n = 13). No difference in aneurysm size [8.1 mm (±4.5) vs. 8.8 mm (±8.1); p = 0.6) was seen between the coiling and PED groups. No difference in ASA grade (p = 0.5) was observed, as most patients were either grade 2 (5.4% vs. 20.8%) or grade 3 (16.2% vs. 27.3%) for both coiling and PED, respectively (after excluding patients with missing grades). No difference in discharge disposition was seen (p = 0.1). The mean length of stay was similar for the coiling [3.1 (±5.5) days] and PED [2.4 (±2.6) days; p = 0.2] groups. The mean duration of follow-up was also similar for the coiling [22.7 (±18.5) months] and PED [18.6 (±14.9) months; p = 0.2] groups. No difference in the length of clinical (p = 0.9) or imaging (p = 0.5) follow-up was seen between the groups.

**Table 1:** Baseline characteristics of 114 patients with unruptured aneurysms Results are presented as standard deviations of the mean (±) or absolute numbers with the percentage of the total P-value: probability value; ICA: internal carotid artery; ACA: anterior cerebellar artery; PComm: posterior communicating artery; AComm: anterior communicating artery; MCA: middle cerebral artery; Vert/PCA/SCA/PICA/AICA: vertebral artery/posterior cerebellar artery/superior cerebellar artery; posterior inferior cerebellar artery/anterior inferior cerebellar artery; ASA: American Society of Anesthesiologists; SNF: skilled nursing facility

Variable	Coiling (N = 37)	Pipeline (N = 77 patients, 79 aneurysms)	P-value
Age (years)	61.3 (±12.8)	57.0 (±14.5)	0.2
Sex (male)	12 (32.4%)	17 (22.1%)	0.2
Aneurysm location			0.0001
ICA	7 (18.9%)	51 (64.6%)	
ACA	4 (10.8%)	3 (3.8%)	
Acomm	11 (29.7%)	1 (1.3%)	
Pcomm	3 (8.1%)	3 (3.8%)	
MCA	0	4 (5.1%)	
Basilar	5 (13.5%)	4 (5.1%)	
Vert/PCA/SCA/PICA/AICA	3 (8.1%)	13 (16.5%)	
ICA + Vert	1 (2.7%)	0	
ACA + PComm	1 (2.7%)	0	
Unknown	2 (5.4%)	0	
Aneurysm size (mm)	8.1 (±4.5)	8.8 (±8.1)	0.6
ASA grade			0.5
1	0 (0.0%)	1 (1.3%)	
2	2 (5.4%)	16 (20.8%)	
3	6 (16.2%)	21 (27.3%)	
4	2 (5.4%)	3 (3.9%)	
Unknown	27 (73.0%)	36 (46.8%)	
Discharge disposition			0.1
Home	32 (86.5%)	71 (92.2%)	
Home health	0 (0.0%)	2 (2.6%)	
Acute rehabilitation	1 (2.7%)	3 (3.9%)	
SNF	2 (5.4%)	1 (1.3%)	
Died	2 (5.4%)	0 (0.0%)	
Length of stay (days)	3.1 (±5.5)	2.4 (±2.6)	0.4
Length of overall follow-up (months)	30.8 (±24.6)	26.4 (±18.6)	0.3
Length of clinical follow-up (months)	26.0 (±23.5)	23.5 (±18.7)	0.8
Length of imaging follow-up (months)	25.5 (±22.7)	24.4 (±17.4)	0.5

Subcategory costs

**Figure 1:**
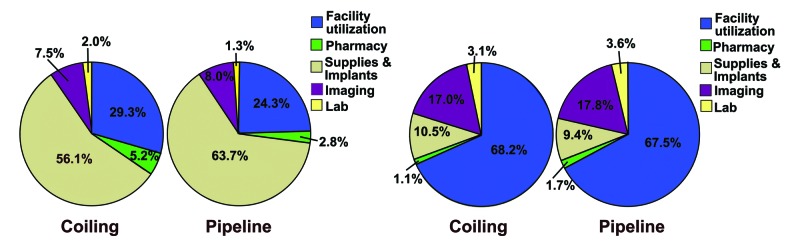
Cost allocation Comparison of cost allocation during hospital stay (left pie charts) and in combined follow-ups (right pie charts)

Subcategory costs were calculated for the initial admission and combined follow-up visits (Figure 1). During the initial admission for coiling treatment, an average of 56.1% of the total cost was found to be related to supplies and implants and 29.3% was for facility utilization. For PEDs, an average of 63.7% of the total cost was supplies and implants and 24.3% was for facility utilization. In both cases, the follow-up costs were predominantly associated with facility utilization (68.2% of the total cost for coiling and 67.5% for PEDs). Overall, there were no differences in cost distribution at the time of treatment (chi-squared, p = 0.9) or follow-up (chi-squared, p = 0.95).

Total cost

**Figure 2:**
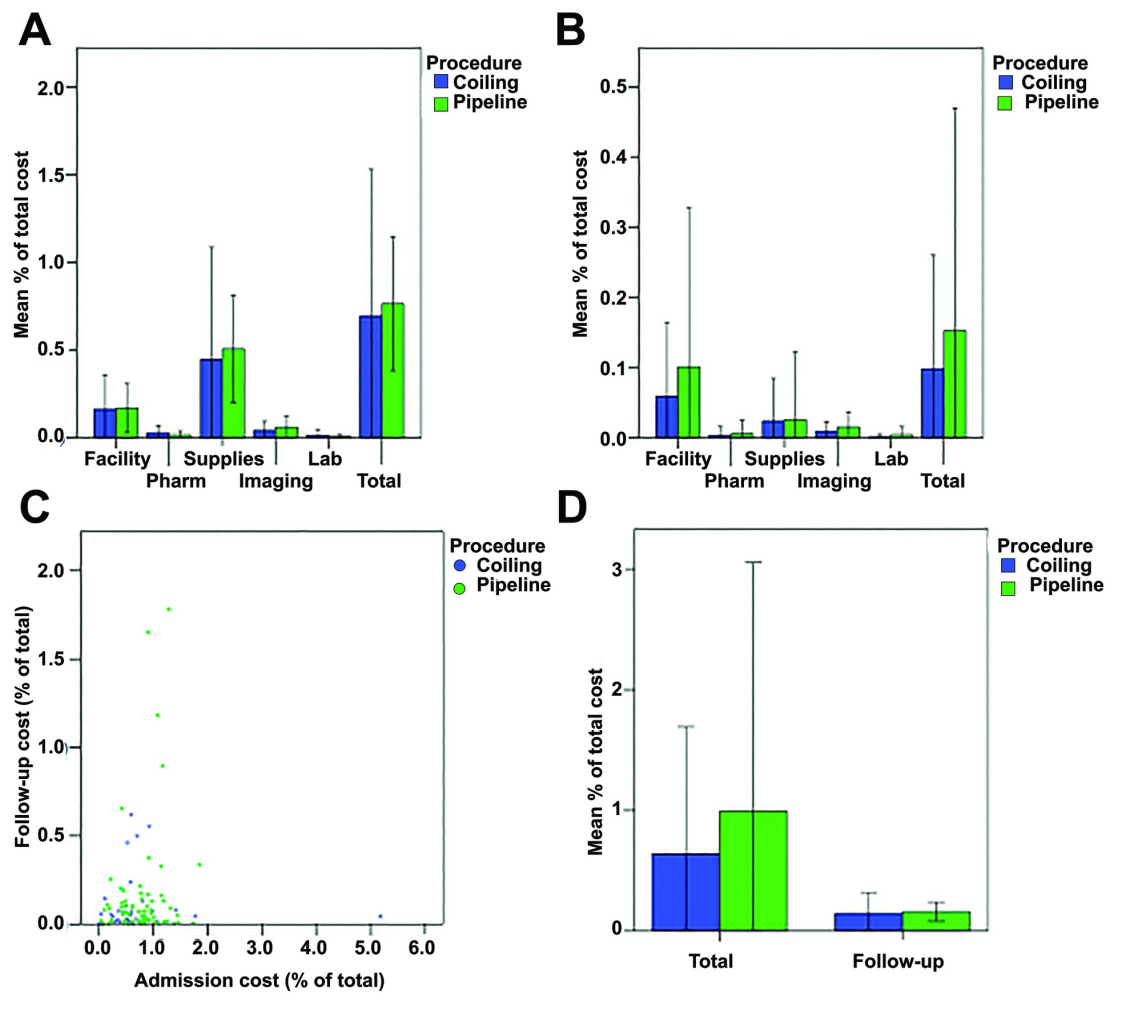
Evaluation of average cost A) The mean costs of the initial hospital stay were not significantly different between coiling and PED treatments for facility (p = 0.9), pharmacy (p = 0.06), supplies/implants (p = 0.5), imaging (p = 0.1), laboratory (p = 0.2) or the entire subcategories (p = 0.5). B) Similarly, follow-up mean costs were similar for facility (p = 0.3), pharmacy (p = 0.6), supplies/implants (p = 0.9), imaging (p = 0.1), laboratory (p = 0.2), or the entire subcategories (p=0.3). C) The regression correlation of admission costs and follow-up costs was poor (r = 0.1, p = 0.3). There were several outliers for either the admission or follow-up costs. D) The magnitude of follow-up costs was smaller than that of the initial costs for both coiling and PED treatments

A comparative analysis of the average patient costs is shown in the figure below (Figure 2). For the initial admission, there was no difference between coiling and PED in the subcategory (p-value range: 0.06-0.9) or the total cost (p = 0.5) (Figure 2 A). Large, overlapping error bars suggested significant variability in costs. Similarly, there was also no difference between coiling and PED in the subcategories (p-value range: 0.1-0.9) and total (p=0.3) costs in follow-up (Figure 2 B). The follow-up costs were greater than 10% of the initial costs on average and shifted from predominantly supply costs in the initial treatment to facility costs during follow-up. A comparison of the initial and follow-up costs showed a poor correlation (r = 0.1, p = 0.3) (Figure 2 C). There were several outliers in terms of admission or follow-up costs. Shown differently, the follow-up costs represented a much smaller portion compared with initial admission costs (Figure 2 D).

## Discussion

Study findings

In this study, we found that the upfront and follow-up costs are mostly similar for the treatment of intracranial aneurysms irrespective of whether the providers used coiling or PED methods. A variety of aneurysm locations were analyzed, allowing for a real-world comparison of endovascular-technique costs. A shift from a predominance of costs from supplies/implants upfront to dominance of facility costs during the follow-ups was observed for both coiling and PED treatments, with short-term follow-up cost accounting for a much smaller fraction (<10%) of overall patient treatment costs. The cost of PED was not significantly greater than coiling either at initial treatment or during follow-ups. This study supports a cost-neutral approach towards endovascular treatment, favoring neither PED nor coiling, which may better inform how treatment selection can be done in a cost-conscious manner.

Follow-up endovascular costs

Previous studies of endovascular-treatment costs have presented conflicting findings because of the diversity of aneurysm characteristics and treatment types. Coil embolization of ruptured aneurysms is associated with lower upfront costs and follow-up complication rates compared with microsurgical clipping in some parts of the world (e.g., South Korea) [20]. However, in two recent meta-analyses, follow-up costs in ruptured [20] and unruptured [21] aneurysms in the US were found to be similar regardless of whether clipping or coiling treatments were employed. One criticism of coiling for treatment of ruptured aneurysms has centered on the potential for rebleeding and retreatment; however, large-scale studies have shown that rebleeding rates after coiling have been low: 0.11% (CARAT study) [4], 0.08% (ISAT study) [6], and 0% (BRAT; 6% retreatment rate) [2]. The efficacy of flow diversion for the treatment of previously uncoilable intracranial aneurysms was demonstrated in the seminal pipeline for uncoilable or failed aneurysms (PUFS) trial, which showed good outcomes with the use of PED from both aneurysm-occlusion and safety standpoints [7]. A high rate of aneurysm occlusion (79/91 patients) and low risk of safety endpoint (e.g., major stroke or death; 6/107 patients) were observed at a 1-year follow-up. The follow-up of the PUFS trial at 3 years showed aneurysm occlusion in 78 of 107 patients and primary safety endpoints of 6 in 107 patients [14], results that have been supported by other groups [22-27]. Several studies have compared treatment costs for ruptured aneurysms undergoing PED and those undergoing coiling [16,20]; however, comparability remains limited because of variation in the types of aneurysms selected for either procedure and the impact of postoperative care in ruptured aneurysms. Besides, very few reports evaluating PED costs are currently available.

Direct cost comparison of PED and coiling has been attempted in several studies [16,25,28,29]. Colby et al. [28] compared 30 PED and 30 non-PED treatments of anterior circulation aneurysms. The authors showed a lower implant cost [13,175 USD (±726) vs. 19,069 USD (±2,015); p = 0.013] and lower overall cost [16,445 USD (±735) vs. 22,145 USD (±2,022); p = 0.02) in the PED group. They argued that a lower cost was seen for PEDs because of the reduced need for multiple coils. Malhotra et al. [25] used a decision-tree analysis to evaluate the cost of unruptured aneurysm coiling and potential follow-up events in a theoretical 50-year-old patient. By using a Markov model with various follow-up scenarios, they found lower overall costs when preventative upfront treatment was limited and with a reduction of follow-up imaging in aneurysms that are <3 mm in size. However, when the aneurysm rupture risk exceeded 1.7% annually, earlier coiling treatment was found to be more effective from an overall healthcare-cost perspective. Several limitations in these studies included heterogeneity of treated aneurysm types (e.g., size, location, rupture status, treatment modality), factoring of upfront risk, limited long-term follow-up, and limited availability of direct financial cost [30]. Our results add to the available literature by suggesting that follow-up costs did not differ between coiling and PED cohorts for the treatment of various unruptured aneurysms; furthermore, these costs were a small fraction of the overall treatment costs.

Limitations

The strength of this study is that it represents an analysis of the real-world costs of aneurysm embolization procedures in a busy neurointerventional practice using a well-documented cost database. The coiling and PED techniques were both established and familiar techniques for the surgeons in this practice, who chose the clinically appropriate treatment for each patient/aneurysm. This was further demonstrated in our study of the similar sizes of aneurysms treated with coiling and flow diversion. One limitation of the study is that it was a retrospective single-center study and follow-up times were relatively short in terms of lifetime risk that an aneurysm presents. Thus, the results may not be fit to be generalized to a wider population, different US regions, or a different healthcare system that may have another type of cost structure. Also, the relatively small number of patients included in the study (N = 114) could result in a type 2 error because of the study being underpowered. Future studies should seek to include a larger population across multiple centers to accommodate these potential sources of bias as well as a longer follow-up period. Besides, the lack of a uniform follow-up protocol for coiled or PED aneurysms may have accounted for the wide variability (i.e., standard deviations) of follow-up costs. Another limitation of cost studies in endovascular treatment is the inability to perform a direct comparison of coiling and PED cases. Our results showed that internal carotid artery aneurysms were predominantly treated by PED while anterior communicating artery locations were primarily treated by coiling. These differences in indications may affect the cost and are difficult to control.

## Conclusions

This study evaluated the upfront and follow-up costs incurred by 37 patients who underwent coiling of aneurysms and 77 patients who underwent PED treatment. Overall, the results of this study demonstrate that the upfront and initial follow-up costs for the treatment of intracranial aneurysms remain broadly similar irrespective of whether the providers used coiling or PED endovascular techniques. Upfront costs were predominantly accounted for by device costs, while follow-up costs mostly involved facility fees. Although previous studies have aimed to evaluate whether one type of endovascular treatment is more cost-effective than another, follow-up costs have rarely been included in the equation. Hence, for a large array of aneurysms over a similar period of time, follow-up costs were not shown to be a definitive factor impacting treatment costs. Our results suggest that follow-up costs may not necessarily be a deciding factor for treatment decisions when considering an average aneurysm.
